# Toxicometabolomics and Biotransformation Product Elucidation in Single Zebrafish Embryos Exposed to Carbamazepine from Environmentally-Relevant to Morphologically Altering Doses

**DOI:** 10.1021/acs.chemrestox.1c00335

**Published:** 2022-02-15

**Authors:** Anton Ribbenstedt, Malte Posselt, Jonathan P. Benskin

**Affiliations:** Department of Environmental Science, Stockholm University, 114 18 Stockholm, Sweden

## Abstract

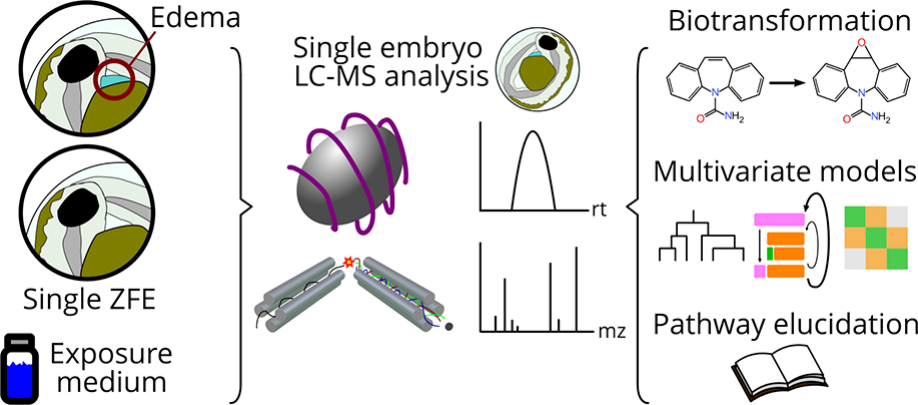

Toxicometabolomics and biotransformation product (bioTP) elucidation were carried out in single zebrafish (ZF) embryos exposed to carbamazepine (CBZ). Exposures were conducted in 96-well plates containing six CBZ concentrations ranging from 0.5 μg/L to 50 mg/L (*n* = 12 embryos per dose). In the 50 mg/L dose group, 33% of embryos developed edema during the exposure (120 hpf), while hatching was significantly delayed in three of the lower-dose groups (0.46, 3.85, and 445 μg/L) compared to the control at 48 hpf. Toxicometabolomic analysis together with random forest modeling revealed a total of 80 significantly affected metabolites (22 identified via targeted lipidomics and 58 via nontarget analysis). The wide range of doses enabled the observation of both monotonic and nonmonotonic dose responses in the metabolome, which ultimately produced a unique and comprehensive biochemical picture that aligns with existing knowledge on the mode of action of CBZ. The combination of high dose exposures and apical endpoint assessment in single embryos also enabled hypothesis generation regarding the target organ for the morphologically altering insult. In addition, two CBZ bioTPs were identified without additional exposure experiments. Overall, this work showcases the potential of toxicometabolomics and bioTP determination in single ZF embryos for rapid and comprehensive chemical hazard assessment.

## Introduction

The pharmaceutical carbamazepine (CBZ) was first marketed in 1962 and is primarily used to treat neurological disorders such as epilepsy, schizophrenia, and bipolar disorder. It is currently among the most prescribed anticonvulsants in the world.^1^ In humans, approximately 87% of CBZ is excreted as bioTPs, including the major pharmacologically active metabolites carbamazepine-10,11-epoxide (CBZ-Ep) and carbamazepine-10,11-*trans* dihydrodiol (CBZ-DH), as well as minor hydroxy, quinone, and glucuronide metabolites.^2^ CBZ is inefficiently degraded during wastewater treatment, and it is estimated that ∼98% of CBZ entering a wastewater treatment plant is released to the environment unchanged.^3^ Given its widespread use and environmental persistence, CBZ can be found ubiquitously in surface water at concentrations ranging from 0.1 to 1100 ng/L.^4−6^

High LC50s for CBZ (i.e., 1.5 to ≥245 mg/L) determined across several species have led some researchers to conclude that CBZ poses minimal risk to the aquatic environment.^6,7^ Nevertheless, its ubiquitous occurrence and sublethal effects at low doses have led to renewed concerns that CBZ exposure in fish could lead to adverse effects on the population level.^6,7^ For example, chronic exposure to 0.5 and 10 μg/L CBZ decreased reproductive output in adult ZF, while similar levels (0.01–100 μg/L) perturbed behavior and reproduction in both ZF embryos and *Daphnia magna*.^8,9^ These data point to the need for further information on biochemical perturbations underlying the MoA of CBZ, particularly at environmentally relevant concentrations. To this end, a recent metabolomic investigation of CBZ exposure in *Daphnia magna* reported a nonmonotonic dose response following exposure to 1.75–14 mg/L CBZ, but these levels are much higher than those typically observed in the environment.^10^ Likewise, Huang et al.^11,12^ reported perturbations in targeted metabolites and gene transcripts following exposure of ZF embryos to environmentally relevant concentrations (3.54–4720 μg/L), but the use of targeted methods and the absence of a link with apical endpoints precluded the identification of metabolites that could be linked to an adverse effect. Clearly, further information on the MoA of CBZ is needed, particularly at environmentally relevant concentrations.

To address this knowledge gap, we report here on metabolomic perturbations of single zebrafish (ZF) embryos exposed to six different concentrations of CBZ, spanning both environmentally relevant and apical endpoint-inducing concentrations. These data are supplemented with information on the formation of CBZ bioTPs in both exposure water and single embryos. Together, these datasets were used for generating hypotheses for CBZ MoAs in fish over a wide range of doses, enabling the observation of nonmonotonic dose responses while simultaneously measuring individual variability in the biotransformation of CBZ in single ZF embryos.

## Materials and Methods

### Standards and Reagents

All standards used for the identification of nontargeted features and for the targeted analysis of exposure water were purchased from Sigma-Aldrich. This included CBZ, CBZ-D8, 10,11-epoxidecarbamazepine (CBZ-Ep), 10,11-dihydroxycarbamazepine (CBZ-DH), iminostilbene (Imi), aspartate, benzoic acid, betaine, cytidine, γ-aminobutyric acid (GABA), guanosine, histidine, 2-hydroxycinnamic acid, hypoxanthine, taurine, threonine, and tyrosine. Additional standards and reagents are described in detail in previous work.^13,14^ Methanol (MeOH) and acetonitrile (ACN) were HPLC-grade and purchased from Merck (Darmstadt, Germany). Milli-Q water was produced using a Milli-Q Integral 3 and a Millipak Express 40 (0.22 μm) filter (Millipore, Merck, Darmstadt, Germany) measuring <3 ppb of organic matter.

### Dose Preparation

**Figure 1:**
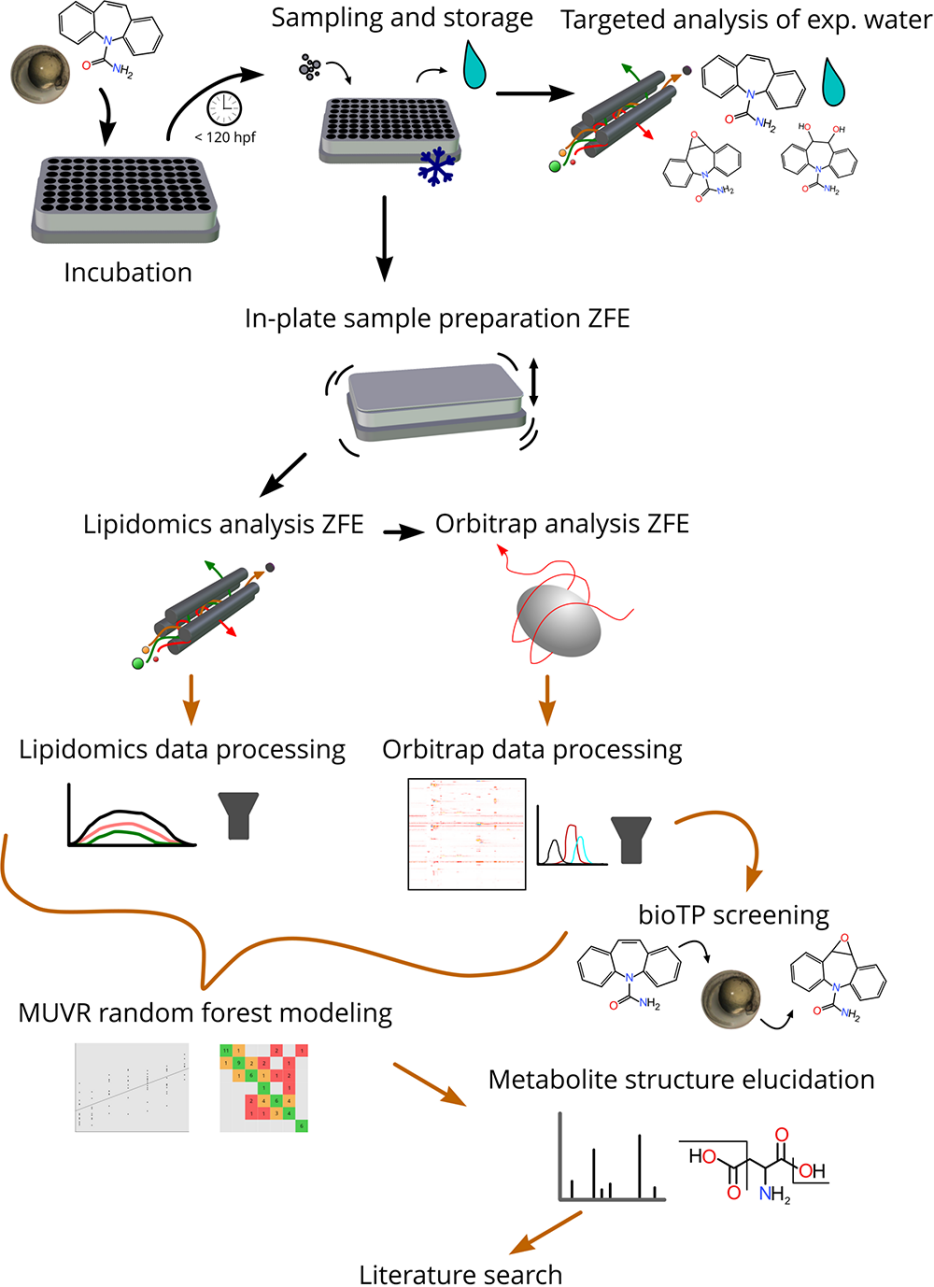
The entire workflow for the paper outlined from beginning to end. Black arrows signify sample preparation and instrumental analysis, while brown arrows represent data processing and analysis.

Concentrations of CBZ in surface water range from 0.1 to 1100 ng/L.^4−6^ Prior toxicity testing in ZF embryos produced an LC_50_ of >245 mg/L, no-observable-effect concentrations (NOECs) of 25 and 30.6 mg/L (developmental effects and mortality, respectively), and a lowest-observable-effect concentration (LOEC) of 50 mg/L (mortality).^7,15^ Beyond ZF embryos, LOECs as low as 0.001 mg/L were observed for antioxidant responses in the muscle of rainbow trout (*Oncorhynchus mykiss*) following a 42 day exposure to CBZ.^16^ Since our goals were to investigate metabolomic perturbations at low doses while also measuring potential transformation products, the following dosing regimen was established, covering both environmentally relevant concentrations and levels previously linked with apical endpoints: 0.5 μg/L, 5 μg/L, 50 μg/L, 500 μg/L, 5 mg/L, and 50 mg/L (nominal). The exposure medium was prepared by mixing CBZ directly in tank water from the SciLife facility (see Table S1 for water parameters). Doses received by each embryo were confirmed following exposure (see the “Analysis of Exposure Medium” section and Figure 1).

### Exposure and In-Plate Mortality Assessment

The ZF embryos in this study were excess material produced by animals used under permit C161.14 from the SciLife Laboratory Zebrafish Facility in Uppsala, Sweden. All experiments were terminated prior to 120 hpf, and therefore, under EU Directive 2010/63/EU, the assays were classified as in vitro and no ethics approval was required.^17,18^ The incubation and microscopy of the embryos were based on OECD test guideline (TG) 236 with deviations noted in the Supporting Information.^19^ Briefly, fertilized embryos collected after spawning were washed and transferred to their respective exposure mediums (i.e., within 20 min of spawning; see Figure 1). Fertilized embryos were then moved to separate wells in a 96-well plate (wp) that had been pre-exposed to the respective doses of CBZ for 24 h. Each 96-wp contained six concentrations of CBZ (*n* = 12 embryos per dose; see Table S2), tank water (negative control; *n* = 12 embryos), and 3,4-dichloroaniline (positive control; 4 mg/L; *n* = 12 embryos).

Embryos were inspected at 48 and 120 hpf for lethal and sublethal apical endpoints described in the TG 236 guideline and Nagel et al.^20^ Just prior to 120 hpf, the incubation water was transferred to an empty 96-wp, which was subsequently frozen for future dose characterization, while the embryos were terminated through freezing on dry ice prior to transport to Stockholm University, where they were maintained at −80 °C prior to extraction.

### Analysis of Exposure Medium

Following termination of the experiments, concentrations of CBZ and the bioTPs CBZ-Ep and CBZ-DH were quantified by diluting the exposure medium with MeOH containing an internal standard (IS) and then analyzing the diluted medium by liquid chromatography tandem mass spectrometry (LC–MS/MS) using an Ultimate 3000 ultrahigh-performance liquid chromatograph (UHPLC; Thermo, USA) coupled to a TSQ Quantiva triple quadrupole mass spectrometer (Thermo, USA) as described previously.^21^ Further details are provided in the Supporting Information. Measured CBZ doses following exposure were 43,367, 4854, 445, 41, 3.85, and 0.46 μg/L, with RSDs ranging from 4–12% (see Table S2), which are within 77–97% of the nominal. The increasing amount of dilution required to measure CBZ in successively higher doses resulted in the intermittent quantification of only CBZ-Ep (in the 43,367 and 445 μg/L doses) despite the fact that CBZ-Ep and CBZ-DH were above detection limit in most doses (following exposure).

### Analysis of ZF Embryos

The embryos were prepared through an in-plate extraction method described previously.^14^ In short, 120 μL of a MeOH:chloroform (80:20) mixture containing an IS was added to each well along with mixed-size stainless steel beads. A silicone lid with a polytetrafluorethylene surface layer was glued onto the plates prior to homogenization, sonication, and centrifugation. Following extraction, the samples were subjected to both targeted lipidomics and nontarget metabolomics analysis.^13^ For the lipidomics analysis, plates were fitted directly into the autosampler of the same UHPLC–MS/MS system used for dose characterization (see previous section). After the flow-injection lipidomics analysis, the plate was moved to the autosampler of another Ultimate3000 UHPLC fitted with a hydrophilic interaction liquid chromatography (HILIC) column (BEH amide; Waters, USA) connected to a Q Exactive Orbitrap HRMS (Thermo, USA) via an electrospray ionization source. The instrument was operated in positive mode utilizing nontargeted full scan acquisition with data-dependent MS2 analysis of the three highest-intensity features.

### Quality Control

In both methods described above, a quality control (QC) sample was run every 10th (lipidomics method) or 5th (orbitrap method) injection to monitor (and ultimately correct for) sequence drift. The small final sample volume (∼120 μL) and the large injection volumes necessitated preparation of the QC samples using pooled embryos (*n* = 10) in an Eppendorf tube. This tube was prepared concurrently with and using the exact same procedures as for those prepared in the 96-wps. A separate blank plate was also prepared concurrently with the exposure plate in order to identify any background contamination from the procedure itself.

### Metabolomic Data Processing

Targeted lipidomics data processing involved the integration of raw data using XCalibur 3.0.63 (Thermo, USA) and importing the data into R for batchCorr sequence correction and mass deconvolution as described in detail elsewhere.^13,14^ After IS normalization and removing features displaying a ratio between blanks and negative controls of <10, a total of 102 lipids remained. Nontarget metabolomic data were preprocessed using Compound Discoverer 3.1 (CD; Thermo, USA), which included peak picking, isotope pattern matching, retention-time alignment, gap-filling, and compound grouping. Following preprocessing, a total of 2398 raw features were obtained. Endogenous metabolites were separated from exogenous substances (including bioTPs, in-source fragments, blank contamination, etc.) using a combination of the R packages ExpMetFilter, BatchCorr, and ramclustR.^14,22^ Briefly, the workflow consisted of (i) removal of features that were absent in the QC (e.g., exposure compound, related impurities, and bioTPs), (ii) sequence drift correction and removal of features in the QC with >30% RSDs after correction, (iii) removal of noise and blank features (i.e., features that did not exceed a certain threshold), (iv) detection and removal of in-source fragments of the exposure compound and potential bioTPs predicted by CD, (v) removal of features within a mass error of 5 PPM of a list of CBZ bioTPs predicted by CD (see the “bioTP Identification” section for details), (vi) removal of negative intensity features caused by overcorrection of signal drift for some features, and (vii) removal of features in blanks and QC blanks occurring at >40% of the area of the maximum sample or QC peak, respectively. In total, 598 metabolite features from the combined datasets were used for toxicometabolomic multivariate statistical modeling.

### bioTP Identification

BioTPs were identified using a previously developed data analysis workflow.^23^ Briefly, features that were matched to bioTP exact masses predicted by CD (see Figure 1) were selected for further investigation. Thereafter, a modified version of the previously described filter from step (i) of the metabolomic data processing workflow was applied, which removed features with a signal in the negative control samples (*n* = 12). Any remaining features with MS2s were imported into Sirius+CSI:FingerID (hereon referred to simply as Sirius) for structural prediction.^24^

### Toxicometabolomic Model Development and Pathway Analysis

**Figure 2:**
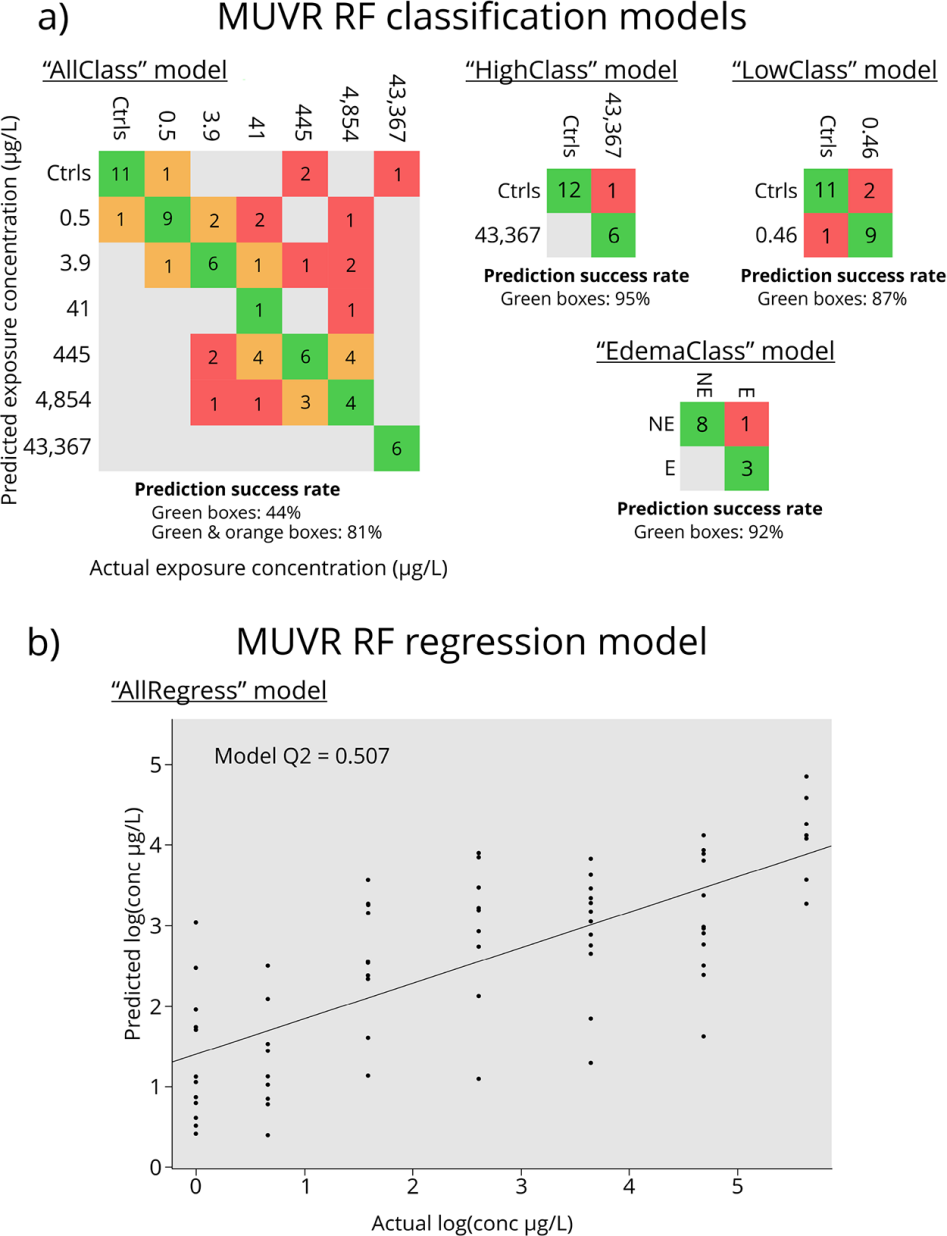
Results from MUVR random forest (RF) modeling of the metabolite features. (a) All four classification models with the predicted dose on the *y* axis and the actual dose on the *x* axis. Values indicate the number of embryos, green boxes indicate correct classification, orange boxes indicate classifications within one order of magnitude of the correct dose, and red boxes indicate classifications outside of one order of magnitude of the correct dose; E = edema, NE = non-edema. (b) Regression model of all six doses and negative controls with predicted log concentration on the *y* axis and actual log concentration on the *x* axis.

Multivariate statistical analysis of the features determined to be endogenous metabolites through our filters was accomplished using the R package MUVR, which performs minimal variable selection through recursive variable elimination by repeated double cross-validation (see Figure 1 and Table S3).^25^ In order to obtain a comprehensive assessment of the effects of CBZ exposure on the ZF embryo metabolome, the following five MUVR random forest models were developed (see Figure 2).

(1) “AllRegress”: A regression model of all six exposure doses of CBZ, as well as negative controls, which aimed to capture metabolites exhibiting a monotonic dose response. Embryos exhibiting lethal and sublethal apical endpoints were removed.
(2) “AllClass”: A classification model for metabolites consisting of all six exposure doses and negative controls aimed for the detection of metabolites with nonmonotonic dose responses. Embryos exhibiting lethal and sublethal apical endpoints were removed.
(3) “HighClass”: A classification model for the highest dose (i.e., 43,367 μg/L) and negative controls aimed at capturing metabolites only relevant for higher-exposure doses.
(4) “LowClass”: A classification model that distinguishes between controls and low environmental concentration exposure (0.46 μg/L) exclusively based on these groups.
(5) “EdemaClass”: A classification model that only considers embryos with and without edema in the highest dose (43,367 μg/L).

### Metabolite and bioTP Identification

Structural elucidation was attempted for metabolites elected by at least one of the five aforementioned models. To predict the molecular structure of both endogenous metabolites and potential CBZ TPs from MS2-data, a combination of Sirius, mzCloud, and fragment ion search (FISh) scoring was used (see Figure 1 and Table S4). Both Sirius and FISh scoring perform in silico fragmentation of a structure, but while FISh makes a biased comparison to fragments of a user-supplied structure, Sirius is unbiased, predicting molecular moieties based on the fragmentation and then comparing to structural databases. mzCloud, on the other hand, compares experimentally derived MS2s to an online library of spectra measured from standards. When the majority of structural predictions (based on MS2 spectra from individual samples) made by Sirius agreed with each other and with mzCloud, we obtained standards for structural verification. Two separate analytical standards were prepared, one for CBZ TPs and one for endogenous metabolites, containing all predicted substances that were available (see list in the “Standards and Reagents” section) and run with the identical instrumental setup as the samples. The MS2 spectra from the standards and samples were then compared using the R-Script NTScreener, which produces a similarity score between 0 (different) and 1 (identical) based on how many of the fragments are shared and how well their intensities are correlated.^26^ All compounds with a similarity score of above 0.9 were considered to be identified at confidence level (CL) 1 according to the Schymanski scale.^27^

## Results and Discussion

### Apical Endpoints

At 120 hpf, mortality in positive controls was 92% (*n* = 11 embryos). No mortality was observed in negative controls or dosed embryos with the exception of the 50 μg/L (25% mortality (*n* = 3 embryos)) and 500 μg/L (8% mortality (*n* = 1 embryo)) dose groups (Table S5). Interestingly, hatching was significantly delayed in three of the lower-dose groups (0.46, 3.85, and 445 μg/L) when compared separately against the control (all *p* = 0.041) at 48 hpf. Sublethal apical endpoints (as defined by the FET assay and Nagel et al.^20^) were only observed in the 43,367 and 0.46 μg/L dose groups and positive controls (33% (*n* = 5 embryos), 8% (*n* = 1) and 8% (*n* = 1), respectively), where the endpoint was pericardial edema for all but one of the embryos.^20,28^ This corresponds well to the findings of Pohl et al.,^29^ in which embryo toxicity (mostly pericardial edema) was observed in 88% (*n* = 14) of ZF embryos (144 hpf) exposed to 30,000 μg/L CBZ and 100% of ZF embryos in the 50,000 μg/L treatment group, while another study by van Woudenberg et al.^30^ reported a delayed onset of hatching (72 hpf) and pericardial edema (96 hpf), resulting in EC_50_s of 45,500 and 52,000 μg/L, respectively.

### Statistical Modeling and Metabolite Identification

All five models were statistically significant; however, after closer inspection, the low-dose model was discarded due to the low number of metabolite features elected by the model (*n* = 4) and because only one of four features were biochemically meaningful. The “AllRegress” model (based on all six doses and negative controls) had a Q2 value of 0.51 and was statistically significant (*p* = 1.6 × 10^–8^, *n* = 100 permutations). The “AllClass” model was highly significant (*p* = 9.2 × 10^–10^, *n* = 100 permutations) and had a misclassification rate of 42%. However, when restricting misclassifications to doses over an order of magnitude apart, the rate was only 19% (see Figure 2). The remaining classification models (“HighClass” and “EdemaClass”) were also statistically significant (*p* = 0.003–0.0008; *n* = 100 permutations per model) and showed low rates of misclassification (5–9%).

Of the 569 metabolite features initially obtained from data filtering, 89 were significant from at least 1 of the models, including 22 from lipidomics (CL 2–3; Table S6) and 67 from nontarget analysis. Initially, 15 of the 67 nontarget metabolite features were identified at CL 1 with authentic standards, but four of these were attributed to two metabolites ([M + H] and [M + K] adducts of cytidine and [M + H] and [M + Na] adducts of histidine), resulting in a total of 13 unique metabolites confirmed at CL 1. Among the remaining 52 metabolite features, two had mzCloud scores of >90% and were considered to have been identified at CL 2. Sirius predicted the structure of 29 of the remaining 50 metabolite features, giving them a CL of 3–4. Of the remaining 21 features, two contained multiple MS2 spectra for which all Sirius-predicted chemical formulae agreed and were thus considered to have been identified at CL 4. Among the other 19 features, three appeared exclusively in QCs (i.e., not in any of the embryos) and were therefore removed, while some had inconsistent predictions by Sirius or mismatching chemical formulae with CD and the rest did not generate any MS2s, disallowing any identification at a CL higher than 5.

### Identification of CBZ bioTPs

**Figure 3:**
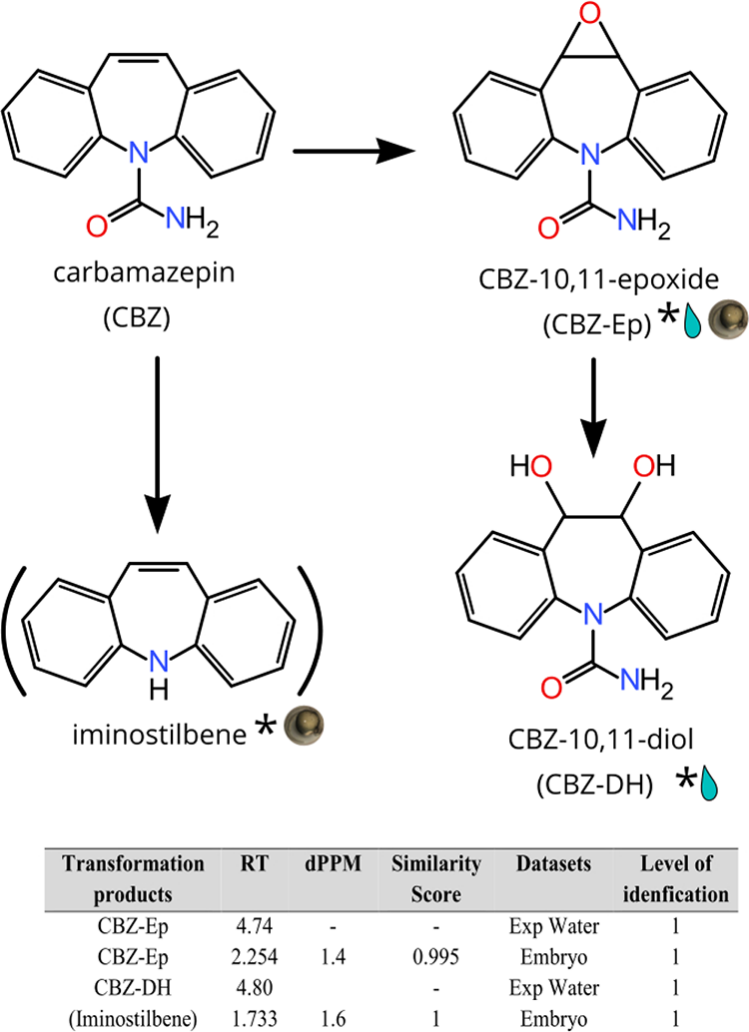
CBZ bioTPs detected in ZF embryos (denoted by embryo marker) or exposure water (denoted by water droplet). Asterisks denote CBZ bioTPs identified at CL 1. Structures within parentheses could not be ruled out as in-source fragments.

ExpMet-filter matching of predicted CBZ bioTP exact masses with the features obtained from CD resulted in only 12 matches (see Table S7). Out of these 12, only five had MS2 spectra, totaling five features of interest possible to identify at a CL better than 5 (see Figure 3 and Table S7). Using native standards, two of these five features (CBZ-Ep and iminostilbene) were elucidated at a CL of 1. However, iminostilbene could not be ruled out as an in-source fragment of CBZ. For the remaining three features, none of the chemical formulae or structures predicted by Sirius showed any resemblance to the bioTPs suggested by CD. Overall, the bioTP results reported here are in agreement with the findings of Jeon and Hollander,^31^ who carried out CBZ bioTP screening in S9 extracts of trout (*Oncorhynchus mykiss*) liver and found a single CBZ bioTP, CBZ-Ep. They also noted that CBZ altered the biotransformation of other compounds when evaluated together. Interestingly, we were able to detect a second bioTP of CBZ, CBZ-DH, through analysis of the exposure medium. CBZ-DH is formed from CBZ-Ep, facilitated by soluble epoxide hydrolase (sEH) enzymes, and has been previously reported to form in fish.^32,33^ One of these studies found sEH in high quantities in the liver, kidney, and gills of rainbow trout (*Salmo gairdneri*). The discrepancy in CBZ-DH formation could indicate that epoxide hydrolase activity is low or nonpresent in liver microsome extracts, possibly through sEH inactivation during the preparation of the microsome extract because CBZ-DH is predominantly biotransformed in the gills, kidneys, or a combination of the two. Regardless of the underlying reason, this illustrates an interesting benefit of using ZF embryos over more conventional (i.e., cell homogenate-based) in vitro tests.

### CBZ Mode of Action

#### Phosphatidylcholine and Choline Metabolism

**Figure 4:**
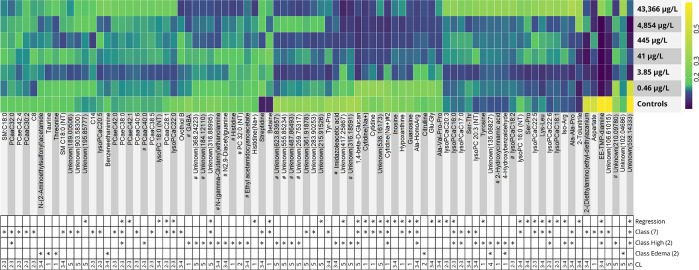
Heatmap of average internal standard-normalized signal intensity over the six doses for the metabolite features elected by all three random forest MUVR models. Each line was internally rescaled to the top intensities in order to more clearly visualize the changes over the doses. CL, confidence level; Class (2), classification model of negative controls and highest dose; Class (7), classification model of negative controls and all six exposure doses; Regression, regression model of negative controls and all six exposure doses.

**Figure 5:**
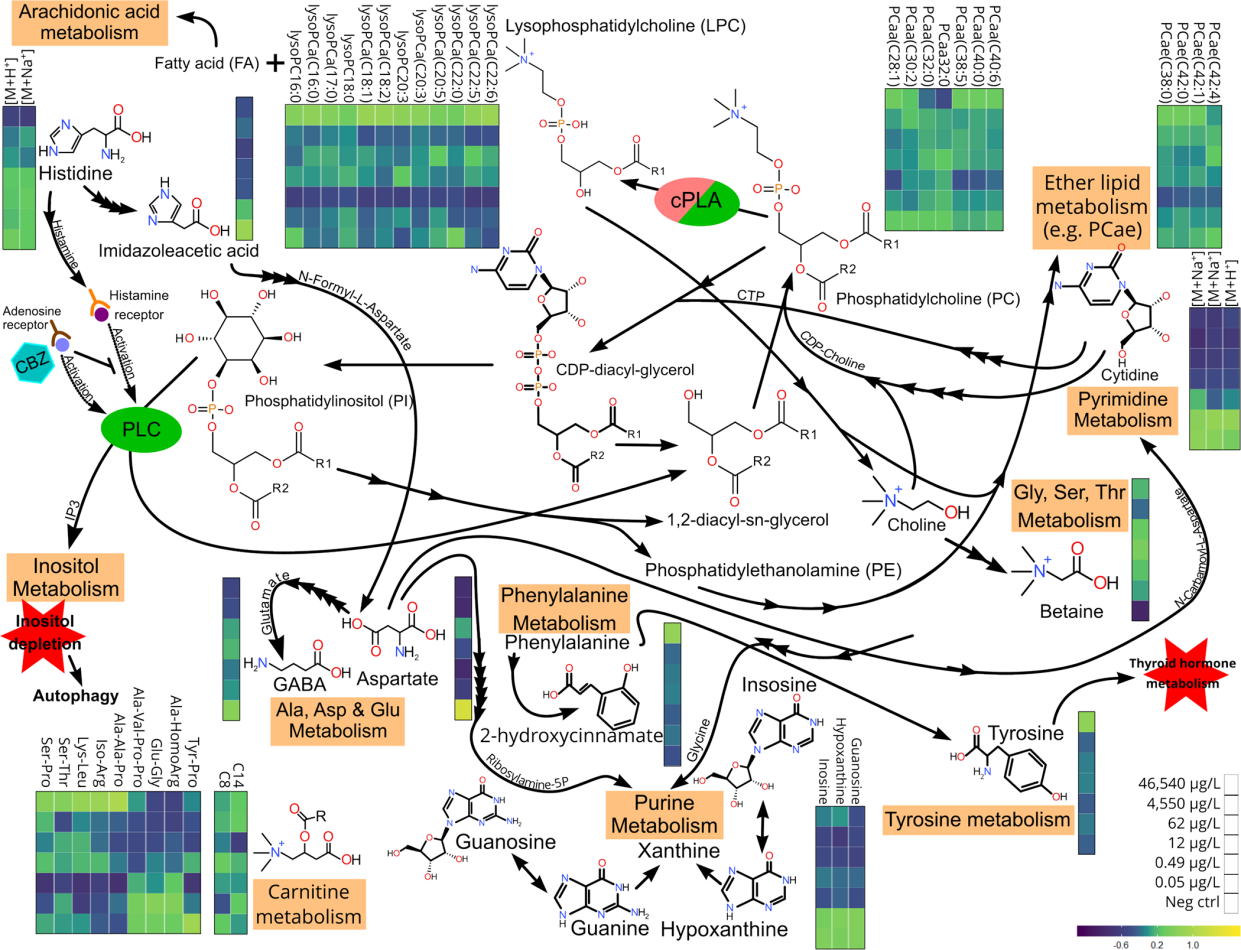
Literature-anchored pathway analysis based off of all confidence level 2–3-identified metabolites elected by the three models. Ovals represent enzymes documented in the literature to be modulated by CBZ exposure (green, upregulated/activated; red, downregulated; and green and red, unclear interaction). Every arrow represents an enzyme that acts on the metabolite, with the name on the arrow being the final metabolite linking into the pathway/prior to becoming the metabolite at the destination of the arrow. Color schemes were taken from the heatmap in Figure 4 and represent relative internal standard-normalized signal intensity.

CBZ has been shown to alter the expression of the enzyme cytosolic phospholipidase 2 (cPLA2), which facilitates PC hydrolysis into fatty acids (FAs) and lysophosphatidylcholine (lysoPCs).^34,35^ In this study, the relative abundance of several PCs and lysoPCs increased considerably in the highest dose (see Figures 4 and 5; 43,367 μg/L), likely reflecting cPLA2-induced compositional changes of PCs. A large increase in the relative abundance of lysoPCs was also observed in the highest exposure dose of CBZ in ZF larvae by Huang et al.^11^ Choline is a component of lysoPCs, a connector between many metabolic pathways, and can be metabolized into betaine (which increased with dose in our study). The purine metabolism pathway is home to many of the putative metabolites elected by our models (see Figures 4 and 5) and has metabolical ties to betaine. Cytidine, which is connected to choline through the metabolite cytidine-triphosphate, was one of the major constituents of the regression model and saw a steady decline with increasing doses (see Figures 4 and 5). The concomitant decrease in cytidine, increase in betaine, and general increase in PCs and lysoPCs with increasing doses in the present work (see Figures 4 and 5) would suggest that increased cPLA2 activity caused by CBZ leads to an increase in choline, which will perturb many of its connected pathways. Previous studies have reported increased levels of acetylcholine as a response to CBZ exposure, which could also be explained by an increased release of choline from lysoPC metabolism.^36,37^

#### Phosphatidylinositol and Histamine Metabolism

The lipid phosphatidyl-1d-myo-inositol directly links glycerophospholipid metabolism and inositol phosphate metabolism and can be further metabolized into free inositol.^38^ It has been shown that inositol depletion is one of the many plausible MoAs of the mood-stabilizing therapeutic effects of CBZ.^39^ Inositol depletion also causes autophagy in cells, which is another documented effect of CBZ.^40,41^ This could explain the marked increase of some di- and tripeptides in the highest-exposure dose of our study (see Figures 4 and 5). Histidine was also elected by the models and interacts with the inositol phosphate metabolism pathway through its metabolism into histamine. A clear decrease of histidine over increasing doses was observed in our study (see Figures 4 and 5), potentially caused by the CBZ induction of adenosine receptors.^42^ Imidazoleacetate is metabolized into aspartate through four reactions and could constitute the link between the perturbations we observed in the histidine and alanine and aspartate and glutamate metabolism pathways (see Figure 5).

#### Histamine, Purine, and Aspartate Metabolism

Adenosine–histamine receptor interactions could explain why hypoxanthine, guanosine, inosine, and aspartate were elected by our models (see Figure 5). Furthermore, CBZ inhibits the cAMP formation, which is also metabolized through the purine metabolism pathway.^43^ The amino acid aspartate is also notable since it connects glycerophospholipid metabolism, purine metabolism, histidine metabolism, and pyrimidine metabolism (see Figure 5). This connection also entails the neurotransmitter GABA.^44,45^ Although it is not its primary anticonvulsant MoA, there is evidence demonstrating that CBZ interacts with the release of GABA in neurons and potentiation of the GABA_A_ receptor.^46−49^ The potentiation of the receptor over time would explain the decrease in GABA in the highest doses in our dataset (see Figure 4). A nonlinear GABA response in ZF embryos exposed to CBZ at concentrations comparable to the lower doses of this study (1–100 μg/L) was previously reported.^50^ In that work, an inverted-shaped dose–response curve was observed, as opposed to the u-shaped dose–response curve found in the present study, in a comparable concentration range.

#### Carnitine Metabolism

Two carnitines, C8 and C14, were elected by the models but lacked any clear trend over the doses (see Figures 4 and 5). Several studies have looked at the effects of various anticonvulsants (including CBZ) on free carnitine with conflicting results and conclusions.^51−53^ Although there is inconsistent evidence of changes in free carnitine concentrations during treatment/exposure to CBZ, it is interesting that carnitine was elected by the classification model involving all doses and that the trend in increasing doses was nonmonotonic, thus being in agreement with the general state of the literature (see Figures 4 and 5).

#### Thyroxine Metabolism

CBZ has a well-documented effect on thyroxine plasma levels in humans.^54−56^ Tyrosine is among the metabolites elected by our models and is tightly connected to thyroid hormone synthesis via its metabolic pathway. The relative abundance of Tyrosine increased 2-fold in the highest dose (see Figure 4) compared to controls, most probably reflecting CBZ effects on thyroid metabolism. Moreover, since thyroid metabolism involves the proteolysis of thyroglobulin, it could also be related to changes of some of the di- and tripeptides observed in the higher doses of this study (see Figures 4 and 5). The exact mechanisms of CBZ thyroid hormone alteration remain to be elucidated, but these observations could be of assistance in the design of future studies.

#### Edema-Related Metabolomic Perturbations and Renal Toxicity

One of the documented side effects of CBZ consumption in humans is renal toxicity, which in extreme cases can lead to edema in human subjects.^57,58^ Glomerular filtration occurs early in ZF development; consequently, ZF embryos have become popular in kidney development research.^59,60^ Interestingly, many of the metabolites elected by the edema model (i.e., citrulline, taurine, and threonine; see Figures 4 and 5) are involved in renal processes and disease.^61−64^ Although threonine has not yet been proven to play any significant role in kidney function or disease, a few studies have shown significant changes in threonine concentrations as the result of impaired kidney function.^61,65,66^ There is evidence suggesting that the glycine, serine, and threonine (GST) metabolism pathway is affected in various kidney diseases, which is also a pathway inhabited by many of the metabolites (e.g., betaine purines, tyrosine, and histamine) elected by the other models.^67−73^ These connections suggest that the developing kidney might be one of the target organs of CBZ metabolomic perturbations in the ZF embryos, which could be of interest in future studies on the toxicity of CBZ in fish.

## Conclusions

In the present work, the determination of biotransformation products and toxicometabolomics was carried out in single ZF embryos exposed to CBZ at doses ranging from environmentally relevant to morphologically altering. The observation of both monotonic and nonmonotonic dose responses painted a unique and comprehensive picture of biochemical perturbations, which offers plausible connections between many previously known MoAs of CBZ. Moreover, the combination of single embryos, apical endpoint analysis, and metabolomics could pinpoint the target organ of high dose exposure. Hypothesis generation regarding the localization of the insult is indeed useful when evaluating the toxicity of a previously untested chemical and can ultimately be used to guide more specific, costly, and cumbersome MoA research. In addition, two CBZ bioTPs were identified without additional exposure experiments. The inclusion of exposure water bioTP screening allowed for the detection of the bioTP CBZ-OHx2, which was readily excreted and therefore not detectable in the nontargeted embryo-only analysis. Application of nontargeted methods to exposure water characterization may help to discover additional novel bioTPs. Overall, this work showcases the potential of toxicometabolomics and bioTP determination in single ZF embryos for improved and comprehensive chemical hazard assessment.

## Supporting Information Available

(Table S5) Apical endpoint data for embryos at 48 and 120 hpf; (Table S6) structure elucidation information for metabolites; (Table S7) structure elucidation information for bioTPs (XLSX)
Deviations from TG236 assay; characterization of dose and excreted biotransformation products; bioTP workflow settings in Compound Discoverer; (Table S1) water conditions at the SciLife Laboratory in Uppsala; (Table S2) concentration of CBZ in the exposure plate; (Table S3) meta-information on MUVR RF model settings; (Table S4) settings for Sirius identification (PDF)
