# Supplementary material for: Toxicometabolomics and Biotransformation Product Elucidation in Single Zebrafish Embryos Exposed to Carbamazepine from Environmentally-Relevant to Morphologically Altering Doses
Source: Chem Res Toxicol. 2022 Feb 15;35(3):431–9. doi: 10.1021/acs.chemrestox.1c00335 (PMC8941598; doi:10.1021/acs.chemrestox.1c00335)
Supplement: Supplementary file 2 — tx1c00335_si_002.pdf [file tx1c00335_si_002.pdf]

Supporting Information Document

Toxicometabolomics and biotransformation product elucidation in single zebrafish embryos exposed to carbamazepine from environmentally-relevant to morphologically altering doses

Anton Ribbenstedt[1*], Malte Posselt[1] and Jonathan P. Benskin[1]

[1]Department of Environmental Science, Stockholm University, Sweden

*Corresponding author:
Anton.Ribbenstedt@aces.su.se

Contents

Deviations from the TG236 assay. 2
Characterization of dose and excreted biotransformation products 2
bioTP workflow settings in Compound Discoverer 2
Table S1. The Water conditions at SciLife in Uppsala 11
Table S2. Concentration of carbamazepine in the exposure plate (LOD 0.020 ug/L ; LOQ 0.020 ug/L) ..11
Table S3. Meta-information on the setup of MUVR for generating the three random forest models 12
Table S4. Settings for Sirius identification 12

Deviations from the TG236 assay.

- Exposure concentrations (a wider range with much lower doses were chosen, leading to lower mortality, than expected in the TG236 assay).
- Exposure time (a longer exposure time, 120 hpf instead of 96 hpf, was chosen).
- Water characterization (the96 wp setup as well as instrumental limitations made us unable to document all parameters documented in TG236).
- Solvent concentration (v/v %) of DMSO in the positive control (only) is higher than allowed in TG236 (0.1% DMSO in this study, 0.01% maximum allowed solvent concentration in TG236).

Characterization of dose and excreted biotransformation products

The exposure water from each well was moved to an empty 96 well plate at the end of zebrafish embryo incubation, frozen and stored at -20˚C until analysis. To avoid carry-over samples were diluted either 1:5, 1:50 or 1:100, depending on the expected concentration, directly in LC-vials. This meant that the negative controls were diluted 1:5 times, the lowest exposure concentration 1:50 and the five highest concentrations were diluted 1:100. The internal standard (carbamazepine-D8; CBZ-D8) was spiked to the final samples following dilution, whereafter all samples and calibration standards contained a 20:80 MeOH:MilliQ mixture and 5 ug/mL CBZ-D8. To further avoid carry-over the injection sequence was segmented by expected concentrations and injected in blocks of increasing dose but with randomized sample order within each concentration block. The analysis was carried out using a TSQ-Quantiva (Thermo, USA) and according to a previous study by Posselt et al.[1]

bioTP workflow settings in Compound Discoverer

Search name: CBZ_bioTPanalysis
Search description: Untargeted Metabolomics workflow: Retention time alignment, Component Detection, Grouping, Elemental Composition Prediction, Gap Filling, Hide chemical Background (using blanks), ID using mzCloud (needs MS/MS) and ChemSpider (using Formula); KEGG Pathway Mapping and Differential Analysis (ANOVA, adjusted p-values, fold change, CV, etc.)
Search date: 6/25/2020 12:03:49 PM
Created with Discoverer version: 3.1.0.305

```
[Input Files (0)]
    -->Select Spectra (36)

    [Select Spectra (36)]
        -->Align Retention Times (2)

        [Align Retention Times (2)]
            -->Detect Compounds (3)
            -->Find Expected Compounds (27)
```

[Generate Expected Compounds (32)]
-->Find Expected Compounds (27)

[Detect Compounds (3)]
-->Group Compounds (23)

[Find Expected Compounds (27)]
-->Group Expected Compounds (33)

[Group Compounds (23)]
-->Fill Gaps (21)
-->Search mzCloud (37)
-->Assign Compound Annotations (40)
-->Search Mass Lists (38)
-->Search mzVault (39)
-->Mark Background Compounds (24)
-->Search ChemSpider (10)
-->Predict Compositions (8)

[Group Expected Compounds (33)]
-->FISh Scoring (34)
-->Mark Background Compounds (35)
-->Search mzCloud (37)
-->Search Mass Lists (38)
-->Search mzVault (39)

[Fill Gaps (21)]

[Search mzCloud (37)]

[Assign Compound Annotations (40)]

[Search Mass Lists (38)]

[Search mzVault (39)]

[Mark Background Compounds (24)]

[Search ChemSpider (10)]

[Predict Compositions (8)]

[FISh Scoring (34)]

[Mark Background Compounds (35)]

[Differential Analysis (12)]

Processing node 0: Input Files

Input Data:
- File Name(s) (Hidden):
    D:\Anton\ZFE#2\180927-Carbamazepin\001_QC_BA2.raw
    D:\Anton\ZFE#2\180927-Carbamazepin\002_QC_BA2.raw
    Etc. etc.……

Processing node 36: Select Spectra
----
1. General Settings:
- Precursor Selection: Use MS(n - 1) Precursor
- Use Isotope Pattern in Precursor Reevaluation: True
- Provide Profile Spectra: Automatic
- Store Chromatograms: False

2. Spectrum Properties Filter:
- Lower RT Limit: 0
- Upper RT Limit: 0
- First Scan: 0
- Last Scan: 0
- Ignore Specified Scans: (not specified)
- Lowest Charge State: 0
- Highest Charge State: 0
- Min. Precursor Mass: 0 Da
- Max. Precursor Mass: 5000 Da
- Total Intensity Threshold: 0
- Minimum Peak Count: 1

3. Scan Event Filters:
- Mass Analyzer: (not specified)
- MS Order: Any
- Activation Type: (not specified)
- Min. Collision Energy: 0
- Max. Collision Energy: 1000
- Scan Type: Any
- Polarity Mode: (not specified)

4. Peak Filters:
- S/N Threshold (FT-only): 1.5

5. Replacements for Unrecognized Properties:
- Unrecognized Charge Replacements: 1
- Unrecognized Mass Analyzer Replacements: ITMS
- Unrecognized MS Order Replacements: MS2
- Unrecognized Activation Type Replacements: CID
- Unrecognized Polarity Replacements: +
- Unrecognized MS Resolution@200 Replacements: 60000
- Unrecognized MSn Resolution@200 Replacements: 30000

Processing node 2: Align Retention Times

----
1. General Settings:
- Alignment Model: Adaptive curve
- Alignment Fallback: Use Linear Model
- Maximum Shift [min]: 1.5
- Shift Reference File: True
- Mass Tolerance: 4 ppm
- Remove Outlier: True

Processing node 3: Detect Compounds
----
1. General Settings:
- Mass Tolerance [ppm]: 4 ppm
- Intensity Tolerance [%]: 30
- S/N Threshold: 3
- Min. Peak Intensity: 120000
- Ions: [M+2H]+2; [M+ACN+H]+1; [M+H]+1; [M+H-H2O]+1
- Base Ions: [M+H]+1; [M-H]-1
- Min. Element Counts: C
- Max. Element Counts: C90 [13]C H190 Br3 Cl4 D5 K2 N10 [15]N Na2 O15 P2 S5

2. Peak Detection:
- Filter Peaks: True
- Max. Peak Width [min]: 1
- Remove Singlets: True
- Min. # Scans per Peak: 10
- Min. # Isotopes: 1

Processing node 23: Group Compounds
----
1. Compound Consolidation:
- Mass Tolerance: 4 ppm
- RT Tolerance [min]: 1

2. Fragment Data Selection:
- Preferred Ions: [M+H]+1; [M-H]-1

Processing node 21: Fill Gaps
----
1. General Settings:
- Mass Tolerance: 4 ppm
- S/N Threshold: 8
- Use Real Peak Detection: True

Processing node 37: Search mzCloud
----
1. General Settings:

- Compound Classes: All
- Precursor Mass Tolerance: 10 ppm
- FT Fragment Mass Tolerance: 10 ppm
- IT Fragment Mass Tolerance: 0.4 Da
- Library: Autoprocessed; Reference
- Post Processing: Recalibrated
- Max. # Results: 10
- Annotate Matching Fragments: False

2. DDA Search:
- Identity Search: HighChem HighRes
- Match Activation Type: True
- Match Activation Energy: Match with Tolerance
- Activation Energy Tolerance: 20
- Apply Intensity Threshold: True
- Similarity Search: None
- Match Factor Threshold: 60

3. DIA Search:
- Use DIA Scans for Search: False
- Max. Isolation Width [Da]: 500
- Match Activation Type: False
- Match Activation Energy: Any
- Activation Energy Tolerance: 100
- Apply Intensity Threshold: False
- Match Factor Threshold: 20

Processing node 40: Assign Compound Annotations
----
1. General Settings:
- Mass Tolerance: 5 ppm

2. Data Sources:
- Data Source #1: mzCloud Search
- Data Source #2: Predicted Compositions
- Data Source #3: MassList Search
- Data Source #4: ChemSpider Search
- Data Source #5: (not specified)
- Data Source #6: (not specified)
- Data Source #7: (not specified)

3. Scoring Rules:
- Use mzLogic: True
- Use Spectral Distance: True
- SFit Threshold: 20
- SFit Range: 20

Processing node 38: Search Mass Lists
----

1. Search Settings:
- Mass Lists: Endogenous Metabolites database 4400 compounds.massList
- Mass Tolerance: 5 ppm
- Use Retention Time: True
- RT Tolerance [min]: 2

Processing node 39: Search mzVault
----
1. Search Settings:
- mzVault Library: Custom mzVault Library.db
- Max. # Results: 10
- Match Factor Threshold: 50
- Search Algorithm: HighChem HighRes
- Match Analyzer Type: True
- IT Fragment Mass Tolerance: 0.4 Da
- FT Fragment Mass Tolerance: 10 ppm
- Use Retention Time: False
- Precursor Mass Tolerance: 10 ppm
- Apply Intensity Threshold: True
- Match Ionization Method: True
- Ion Activation Energy Tolerance: 20
- Match Ion Activation Energy: Match with Tolerance
- Match Ion Activation Type: True
- Compound Classes: All
- Remove Precursor Ion: True
- RT Tolerance [min]: 2

Processing node 24: Mark Background Compounds
----
1. General Settings:
- Max. Sample/Blank: 5
- Max. Blank/Sample: 0
- Hide Background: False

Processing node 10: Search ChemSpider
----
1. Search Settings:
- Database(s): KEGG; LipidMAPS
- Search Mode: By Formula or Mass
- Mass Tolerance: 5 ppm
- Max. # of results per compound: 100
- Max. # of Predicted Compositions to be searched per Compound: 3
- Result Order (for Max. # of results per compound): Order By Reference Count (DESC)

2. Predicted Composition Annotation:
- Check All Predicted Compositions: True

Processing node 8: Predict Compositions
----
1. Prediction Settings:
- Mass Tolerance: 4 ppm
- Min. Element Counts: C H
- Max. Element Counts: C90 H190 Br3 Cl4 K2 N10 Na2 O15 P2 S5
- Min. RDBE: -1
- Max. RDBE: 40
- Min. H/C: 0.1
- Max. H/C: 3
- Max. # Candidates: 10
- Max. # Internal Candidates: 200

2. Pattern Matching:
- Intensity Tolerance [%]: 30
- Intensity Threshold [%]: 0.1
- S/N Threshold: 3
- Min. Spectral Fit [%]: 10
- Min. Pattern Cov. [%]: 90
- Use Dynamic Recalibration: True

3. Fragments Matching:
- Use Fragments Matching: True
- Mass Tolerance: 5 ppm
- S/N Threshold: 3

Processing node 27: Find Expected Compounds
----
1. General Settings:
- Mass Tolerance: 5 ppm
- Intensity Tolerance [%]: 40
- Intensity Threshold [%]: 0.1
- SN Threshold: 3
- Min. # Isotopes: 1
- Min. Peak Intensity: 100000
- Average Peak Width [min]: 0

Processing node 33: Group Expected Compounds
----
1. Compound Consolidation:
- RT Tolerance [min]: 0.5

2. Fragment Data Selection:
- Preferred Ions: [M-2H]-2; [M-H]-1; [M-H-H2O]-1

Processing node 34: FISh Scoring
----
1. General Settings:

- Annotate Full Tree: True
- Match Transformations: True
- S/N Threshold: 3
- High Acc. Mass Tolerance: 2.5 mmu
- Low Acc. Mass Tolerance: 0.5 Da

2. Fragment Prediction Settings:
- Use General Rules: True
- Use Libraries: True
- Max. Depth: 5
- Aromatic Cleavage: True
- Min. Fragment m/z: 50

Processing node 35: Mark Background Compounds
----
1. General Settings:
- Max. Sample/Blank: 5
- Max. Blank/Sample: 0
- Hide Background: True

Processing node 32: Generate Expected Compounds
----
1. Compound Selection:
- Compounds: Carbamazepine (C15 H12 N2 O)

2. Dealkylation:
- Apply Dealkylation: True
- Apply Dearylation: True
- Max. # Steps: 1
- Min. Mass [Da]: 120

3. Transformations:
- Phase I:
    Dehydration (H2 O ->)
    Desaturation (H2 ->)
    Hydration (-> H2 O)
    Nitro Reduction (O2 -> H2)
    Oxidation (-> O)
    Oxidative Deamination to Alcohol (H2 N -> H O)
    Oxidative Deamination to Ketone (H3 N -> O)
    Reduction (-> H2)
    Thiourea to Urea (S -> O)
- Phase II:
    Acetylation (H -> C2 H3 O)
    Arginine Conjugation (H O -> C6 H13 N4 O2)
    Cysteine Conjugation 1 (H -> C3 H6 N O2 S)
    Cysteine Conjugation 2 (-> C3 H7 N O2 S)
    Glucoside Conjugation (H -> C6 H11 O5)
    Glucuronide Conjugation (H -> C6 H9 O6)

Glutamine Conjugation (H O -> C5 H9 N2 O3)
Glycine Conjugation (H O -> C2 H4 N O2)
GSH Conjugation 1 (-> C10 H15 N3 O6 S)
GSH Conjugation 2 (-> C10 H17 N3 O6 S)
Methylation (H -> C H3)
Ornitine Conjugation (H O -> C5 H11 N2 O2)
Palmitoyl Conjugation (H -> C16 H31 O)
Stearyl Conjugation (H -> C18 H35 O)
Sulfation (H -> H O3 S)
Taurine Conjugation (H O -> C2 H6 N O3 S)

- Others: (not specified)
- Max. # Phase II: 3
- Max. # All Steps: 5

4. Ionization:
- Ions: [M+H]+1; [M+Na]+1

Processing node 12: Differential Analysis

----

1. General Settings:
- Log10 Transform Values: True

Table S1. The Water conditions at SciLife in Uppsala

**Temperature**	28.7	°C
**pH**	7.58	
**Conductivity**	1206	uS
**TGP**	100.9	%
**Ammonia**	OK	
**Nitrate**	OK	
**Dissolved Oxygen**	3.9	mg/L

Table S2. Concentration of carbamazepine in the exposure plate (LOD 0.020 ug/L ; LOQ 0.020 ug/L)

	Measured Dose										
	43,366 µg/L		4,854 µg/L		445 µg/L		41 µg/L		3.85 µg/L		0.46 µg/L
B1	46796	C1	5163	D1	434	E1	42	F1	4.34	G1	0.48
B2	43633	C2	5370	D2	408	E2	37	F2	3.62	G2	0.47
B3	46253	C3	5466	D3	458	E3	42	F3	3.64	G3	0.43
B4	44879	C4	4722	D4	443	E4	40	F4	NF	G4	0.46
B5	42830	C5	4675	D5	454	E5	41	F5	4.23	G5	0.44
B6	40869	C6	4112	D6	466	E6	42	F6	3.69	G6	0.62
B7	42084	C7	4846	D7	458	E7	41	F7	3.88	G7	0.42
B8	44687	C8	5008	D8	458	E8	45	F8	3.67	G8	0.44
B9	45268	C9	3690	D9	458	E9	41	F9	3.72	G9	0.46
B10	37796	C10	5706	D10	435	E10	42	F10	4.38	G10	0.44
B11	41459	C11	4289	D11	426	E11	38	F11	3.62	G11	0.42
B12	43849	C12	5199	D12	444	E12	45	F12	3.60	G12	0.46
Mean	46536		4549		62		12		0.491		0
RSD	7%		6%		10%		7%		24%		12%
CI95% lower	44231		4388		58		11		0.422		0.43
CI95% upper	48840		4711		66		13		0.561		0.49

Table S3. Meta-information on the setup of MUVR for generating the three random forest models

	MUVR classification (7 classes)	MUVR classification (2 classes)	MUVR regression model
**$scale**	TRUE	TRUE	TRUE
**$nRep**	30	30	30
**$nOuter**	7	7	6
**$nInner**	6	6	5
**$varRatio**	0.9	0.9	0.9
**$DA**	TRUE	TRUE	FALSE
**$fitness**	"MISS"	"MISS"	"RMSEP"
**$method**	"RF"	"RF"	"RF"
**$methParam**			
**$methParam$`ntreeIn`**	150	150	150
**$methParam$ntreeOut**	300	300	300
**$methParam$mtryMaxIn**	150	150	150
**$methParam$robust**	0.05	0.05	0.05
**$ML**	FALSE	FALSE	FALSE
**$parallel**	TRUE	TRUE	TRUE

Table S4. Settings for Sirius identification

General	
Instrument	Orbitrap
MS2 MassDev	5 (ppm)
Consider only formulas in DBs	Bio Database; HMDB; Kegg; Kegg Mine
Possible ionizations	+H; +Na; +K
CSI:FingerID	
Search in DBs	Bio Database; HMDB; Kegg; Kegg Mine
Fallback adducts	[M+H]+; [M+Na]+; [M+K]+

*Settings not mentioned were not changed from the default

References

(1) Posselt, M.; Jaeger, A.; L. Schaper, J.; Radke, M.; P. Benskin, J. Determination of Polar Organic Micropollutants in Surface and Pore Water by High-Resolution Sampling-Direct Injection-Ultra High Performance Liquid Chromatography-Tandem Mass Spectrometry. *Environ. Sci. Process. Impacts* **2018**, *20* (12), 1716–1727. https://doi.org/10.1039/C8EM00390D.
